# Distortions to the passage of time during England’s second national lockdown: A role for depression

**DOI:** 10.1371/journal.pone.0250412

**Published:** 2021-04-20

**Authors:** Ruth Ogden

**Affiliations:** School of Psychology, Liverpool John Moores University, Liverpool, United Kingdom; Unviersity of Sheffield, UNITED KINGDOM

## Abstract

In attempts to control the spread of the Covid-19 virus, many governments have resorted to imposing national lockdowns on their citizens. Previous research has demonstrated the passage of time becomes distorted for many people during these lockdowns. To date, research has only examined how time was experienced early in initial lockdowns. The current study examined whether distortions to the passage of time were also present later into the global pandemic. An online questionnaire was used to collect passage of time judgments for the day, week and 8 month period since the first UK lockdown. In addition, measures of affect, social satisfaction, task-load, compliance and health status were also recorded. The results show that over 80% of people reported experiencing distortion to the passage of time during the second English lockdown in comparison with normal. Depression, satisfaction with social interaction and shielding status were found to be significant predictors of temporal distortion. A slower passage of time was associated with greater depression, shielding and greater dissatisfaction with social interactions. Feeling like it was longer than 8 months since the UK’s first lockdown was associated with greater depression, increased dissatisfaction with social interaction and greater change of life as a result of lockdown. The results suggest that distortions to the passage of time are an enduring feature of lockdown life and that different factors predict temporal experience during different points in lockdown.

## Introduction

The threat posed by the novel coronavirus-19 has forced governments around the world to impose restrictions on the daily lives of citizens in an attempt to control the spread of the virus. Many countries have resorted to “locking-down” residents, placing limits on their opportunity to leave home, go to work and school and socialise with friends and family. Even significant personal events such as weddings and funerals, along with religious and public celebrations have been subject to extreme curtailment. Such profound changes in daily life have altered perceptions of the world around us, and in particular, the subjective speed at which time appears to be passing [1].

Studies conducted in the UK [1], France [2, 3] and Italy [4, 5] indicate that during the first wave of covid-19 lockdowns, there was widespread and significant distortion to the passage of time during the period of lockdown in comparison with normal (i.e. prior to lockdown). In the UK, for example, 80% of participants reporting experiencing time passing at a speed different to normal during lockdown [1]. Lockdown was also associated with a decrease in the ability to keep track of time and an increase in confusion over the days of the week [4].

Whilst distortion to time was widespread during lockdown, the direction of distortion varied between different countries. In France and Italy, participants experienced a global slowing of time during lockdown in comparison with prior to lockdown [2–4]. Slowing was also noted in a separate Italian study which focused on the experience of time during lockdown for people with dementia with Lewy bodies [5]. In the UK however, experience of time was more varied with approximately 40% of people experiencing a speeding up of time and 40% experiencing a slowing of time [1].

### Social effects

Across studies, there were a number of universal factors which were found to influence the subjective speed of the passage of time during lockdown. In the UK [1] and France [2, 3], some of the primary predictors of temporal experience were what may be considered lockdown specific factors such as levels of boredom, sadness and satisfaction with social interaction. In France, for example, time experience was mediated by sadness and boredom, with greater levels of boredom and sadness being associated with a slowing of time. In the UK, satisfaction with social interactions and level of task load were predictive of the subjective speed of the passage of time during the day. Here, lower levels of satisfaction with social interaction and low levels of task load were associated with a slowing of time. Whilst the variables measured by Ogden (2020) [1], Droit-Volet et al., (2020) [2] and Martinelli et al., (2021) [3] were not identical, they may all be considered to be capturing the essence of what it is to be bored. Low task-load, for example, is associated with increased boredom [6], as is low satisfaction with social interaction [7]. Indeed, in the specific case of covid-19 lockdown, when socialisation was so restrictive, dissatisfaction with social interactions may be considered to be a particularly acute indicator of boredom [7].

### Age effects

In the UK [1] and France [2], negative correlations were also observed between the subjective speed of time and age. Increasing age was therefore associated with a slowing of the passage of time during the pandemic. A slowing of time with increasing age is perhaps counter-intuitive, recent studies have indicated that younger and older individuals experience the passage of time similarly [8] and older studies which do show age effects often report that time speeds up with increasing age [9–13]. However, a recent study of the passage of time of elderly people living in nursing homes revealed a slowing of time unique to this group of people in comparison with independently living elderly people [14]. This, coupled with the age effects observed in lockdown, perhaps suggests that the loss of routine and independence associated with residential care and lockdown (elderly people were advised to shield and in many instances were more reliant on relative for shopping ect.) may be the driver of a slowing of time rather than age per se.

### Affective influences

Interestingly, there were a number of factors which may have been expected to be predictive of the passage of time, which were not. For example, clinical studies show that depression is associated with a slowing of time, with periods of depression dragging by [15–17]. Similarly, laboratory studies of the timing of short durations also indicate that anxiety can distort time processing [18]. During lockdown however, levels of depression and anxiety were not themselves predictive of distortions to the passage of time [1–3]. This may suggest that, for studies conducted early in lockdown at least, the effect of enduring state emotions (e.g. depression and anxiety) were perhaps attenuated by measures of factors which changed acutely and immediately in direct response to lockdown, for example, increases in sadness, boredom and social isolation.

### Subsequent lockdowns

Whilst the studies discussed offer clear support for a lockdown induced distortion to the passage of time, they all represent a snapshot of the *immediate* effect of lockdown on temporal experience. Although it was hoped that lockdown restrictions would be short lived, they often ended up lasting for many months and the emergence of a “second wave” of Covid-19 resulted in further lockdown measures being imposed in many countries. However, it is unclear whether the distortions to the passage of time experienced in the first weeks of lockdown would remain further into the pandemic.

As the duration of lockdown has progressed the long-term effects have become more apparent. Health protecting behaviours such as exercise have reduced [19] and negative health behaviours such as maladaptive eating [20, 21] and alcohol consumption [22] have increased. Mental health and wellbeing have also been negatively affected with widespread significant increases in depression, anxiety, isolation, and loneliness [23–28] being reported. There have however also been positive changes reported, for example, working from home is now more normal, and many people report a desire to remain working from home in some capacity beyond the covid-19 crisis [29]. These longer-term changes in lifestyle, mental health and wellbeing may alter the way in which the passage of time is experienced throughout the pandemic. As the familiarity of lockdown life increases, making it seem more normal and less acutely shocking, it is possible that temporal distortions may reduce in prevalence. However, long-term increases in depression and anxiety, coupled with increases in chronic isolation and loneliness, may result in greater levels of emotion induced distortion to the passage of time. To understand the full extent of lockdown on temporal experience it is therefore important to reassess time experience further into the global pandemic.

In England, a second national lockdown was imposed between the 5^th^ of November 2020 and the 2^nd^ of December 2020. The rules were very similar to that of the first lockdown; people should work from home wherever possible, non-essential shops closed, people were required to stay at home wherever possible and household mixing was prohibited indoors in all circumstances and outdoors in most circumstances. However, unlike during the first UK lockdown, schools remained open. England’s second lockdown therefore provided an opportunity to re-examine the experience of time during a national lockdown.

### The current study

The current study therefore sought to capture how the passage of time was experienced during the second English lockdown. Furthermore, the study aimed to establish the factors which distorted time during the second lockdown. A modified version of the online questionnaire used in Ogden (2020) [1] was distributed to capture temporal experience, mood and other lifestyle factors during the second lockdown. The questionnaire contained three passage of judgement time questions; one exploring the passage of time during that day (POTJ-day), a second exploring passage of time over the last week (POTJ-week) and a third exploring the perceived length of the 8 month epoch since the initiation of the first lockdown (POTJ-8 month). In addition to the questions taken from Ogden (2020) [1], participants were also asked to report whether they were currently shielding and to rate the extent to which they were compliant with current covid-19 restrictions.

Despite increases in the familiarity and normality of lockdown life, it was anticipated that there would be significant distortion to the passage of time during the second English lockdown. Given that the second lockdown significantly curtailed opportunities for face-to-face socialisation, it was expected that, as during the first lockdown, reduced satisfaction with social interaction would be associated with a slower passage of time. In addition, because increasing age has been associated with a slowing of time in multiple countries, it was expected that greater age would be associated with a slowing of the passage of time. However, because of the absence of school closures and the increase in desire to work from home, it was unclear whether the association between task-load and distortions to the passage of time observed in Ogden (2020) [1] would be replicated. Finally, it was anticipated that there would be a significant association between depression, anxiety and the passage of time. Although this latter hypothesis contradicts existing findings from research into POTJ during lockdown [1–3], it is possible that these studies failed to observe associations between depression and POTJ because their timings meant that the effects of lockdown on mental health and wellbeing were not fully manifest so early into the first lockdowns. However, because depression has increased substantially in the general population throughout lockdown [23–27] and because pre-lockdown studies show a slowing of time in people with clinical depression [15–18], it was expected that greater depression would be associated with a slowing of the passage of time.

## Method

### Participants

1037 participants were recruited through volunteer sampling via email and social media advertising. 184 were excluded from the study because they failed to answer one or more questions, or because they were not currently residing in England. This left a final sample of 853 participants with complete datasets. Table 1 shows demographic information. The study was approved by Liverpool John Moores University Research Ethics Committee (ref 20/NSP/01) and all participants gave informed written consent. The study was conducted in accordance with the principles expressed in the Declaration of Helsinki.

**Table 1:** Descriptive statistics of the proportion of participants in different demographic groups and the mean POTJ for each group.

	Mean (SD) %	Mean POTJ-day (SD)	Mean POTJ–week (SD)	POTJ-8 months (SD)
*Age (years)*	32.03 (13.55)			
*Young < 26*	51.90	4.22 (1.84)	4.50 (1.87)	2.44 (1.51)
*Middle aged*	43.10	4.20 (1.49)	4.26 (1.67)	2.53 (1.37)
*Older >60*	5.00	4.14 (1.71)	4.24 (1.67)	2.57 (1.43)
*Gender*				
*Male*	27.50	4.23 (1.65)	4.40 (1.76)	2.54 (1.41)
*Female*	72.50	4.20 (1.71)	4.38 (1.79)	2.47 (1.46)
*Cohabitation status*				
*Living alone*	8.21%	4.47 (1.56)	4.39 (1.53)	2.31 (1.30)
*Cohabiting*	91.79%	4.18 (1.70)	4.38 (1.80)	2.50 (1.45)
*Perceived risk*				
*Yes*	11.61	4.25 (1.78)	4.43 (1.86)	2.53 (1.55)
*No*	79.24	4.21 (1.67)	4.39 (1.75)	2.49 (1.43)
*Unsure*	9.15	4.10 (1.74)	4.29 (1.93)	2.38 (1.53)
*Shielding*				
*Yes*	9.26	4.52 (1.98)	4.71 (2.01)	2.41 (1.49)
*No*	90.74	4.17 (1.66)	4.35 (1.78)	2.49 (1.44)
*Employment status*				
*Employed full time*	32.60	4.10 (1.45)	4.46 (1.60)	2.57 (1.40)
*Employed part time*	11.50	4.21 (1.68)	4.26 (1.83)	2.16 (1.34)
*Unemployed looking for work*	1.80	4.27 (1.79)	3.93 (2.12)	2.47 (1.69)
*Unemployed not looking for work*	1.10	3.78 (1.72)	3.22 (1.86)	3.44 (1.51)
*Retired*	2.20	3.63 (1.26)	3.68 (1.64)	2.32 (1.29)
*Student*	45.60	4.16 (1.82)	4.43 (1.84)	2.52 (1.49)
*Disabled*	0.50	3.75 (2.50)	3.75 (2.50)	1.50 (1.00)
*Furloughed		4.34 (2.00)	4.49 (2.00)	2.37 (1.46)
*Depression*	8.57 (5.94)			
*Normal*	59.60	4.35 (1.49)	4.50 (1.58)	2.66 (1.38)
*Moderate*	37.70	4.00 (1.91)	4.22 (2.02)	2.29 (1.53)
*Severe*	2.70	3.83 (2.25)	4.26 (2.14)	1.52 (0.95)
*Anxiety*	4.30 (4.46)			
*Normal*	78.50	4.27 (1.60)	4.41 (1.68)	2.57 (1.42)
*Moderate*	17.70	4.05 (1.90)	4.37 (2.06)	2.25 (1.53)
*Severe*	3.80	3.47 (2.24)	3.94 (2.26)	1.91 (1.45)
*Stress*	8.67 (5.34)			
*Normal*	83.50	4.26 (1.62)	4.45 (1.71)	2.56 (1.43)
*Moderate*	16.50	3.92 (2.00)	4.06 (2.09)	2.09 (1.50)
*Severe*	0	-	-	-
*Task-load*	18.50 (3.66)			
*Social satisfaction*	2.82 (1.17)			
*Physical activity*	2.78 (1.14)			
*Changed routine*	4.22 (1.05)			
*Conformity*	4.09 (.99)			

*Note that furlough refers to a government scheme which provides people with 80% of their normal salary if they are unable to work due to lockdown restrictions.

### Measures

Participants completed an online questionnaire distributed through Qualtrics.com. The questionnaire was released to participants on the 11^th^ of November 2020, 6 days after the commencement of the lockdown and closed on the 30^th^ of November 2020. Participants had therefore experienced between 6 and 25 days of lockdown at the time of participation. The questionnaire was a modified version of that used in Ogden (2020). As in Ogden (2020) demographic information, average level of physical activity, satisfaction with socialisation, perceived risk, passage of time judgements were recorded. Mood was assessed using the DASS-21 [30] and average daily task load was assessed using a modified version of the NASA-TLX [31]. In addition to the questions included in Ogden (2020), participants were asked whether they were shielding, how compliant they were with lockdown restrictions and whether the period of time since the start of the first lockdown felt less than or more than 8 months. Participants took approximately 5 minutes to complete the questionnaire.

#### Demographic questions

Participants stated their age, gender, employment status, whether they were in a high-risk category for Covid-19, whether they were currently shielding and how many people they lived with.

#### Passage of time judgements

The following questions were posed about the daily and weekly passage of time.

1 *“Thinking about today*, *how quickly has time felt like it is passing in comparison with normal (i*.*e*. *before lockdown)*?*”*
2 *Thinking about this week*, *how quickly has time felt like it was passing in comparison to normal (i*.*e*. *before lockdown)*?

Participants responded using the following 7 point Likert scale: 1. Extremely slow, 2. somewhat slower, 3. a little slower, 4. as normal, 5. a little faster, 6. somewhat faster, 7 extremely fast. A higher score therefore indicated a faster passage of time.

3 *“It is 8 months since the UK first went into lockdown*. *It feels like”*

Participants selected one of the following options 1. A lot longer than 8 months, 2. Somewhat longer than 8 months, 3. About 8 months, 4. Somewhat shorter than 8 months, 5. A lot shorter than 8 months.

#### DASS-21

The DASS-21 is a short version of the 42-item Depression Anxiety Stress Scales [30], which measures depression, anxiety, and stress. The 21-item questionnaire contains three, seven item subscales measuring depression, anxiety and stress. For example, for depression “I couldn’t seem to experience any positive feeling at all”, for anxiety “I was aware of dryness of my mouth” and for stress “I found it hard to wind down.” Responses are provided by indicating the severity with which each item reflected the participants experience: (1) did not apply to me at all; (2) applied to me to some degree; (3) applied to me to a considerable degree; and (4) applied to me very much. The DASS-21 is not a diagnostic tool, however, scores from the DASS-21 can be doubled to enable classification as normal, moderate or severe using the following cut-offs: depression; normal < 9, moderate 10–20 and severe > 21, anxiety; normal < 6, moderate 9–14 and severe > 15 and stress; normal < 10, moderate 11–26 and severe > 27. Cronbach’s alpha for the 21 item DASS questionnaire was 0.95.

#### National Aeronautics and Space Administration-Task Load Index (NASA-TLX)

The NASA-TLX [31] is an extremely widely used measure of subjective workload. The NASA-TLX measures subjective workload using six single item questions measuring: mental demands, physical demands, temporal demands, personal performance, effort and frustration. In the current study, a modified version of the NASA-TLX was used to assess the subjective workload of an average day during the period of lock-down. Participants were asked to rate each of the six items, in terms of their average day during the lock-down period, using a 5 point Likert scale in which a high score indicated greater task demands. For example, “*What were the mental demands of your day*?” Cronbach’s alpha for NASA-TXL questionnaire was 0.60 which suggest relatively low reliability.

*Social satisfaction*. To measure social satisfaction participants were asked to rate “Since the Covid-19 lockdown, how satisfied are you with your daily level of social interaction?” using a 5 point Likert scale in which a high score indicated greater satisfaction.

*Physical activity*. To measure physical activity, participants rated “Since the Covid-19 lockdown, how would you describe your level of physical activity? using a 5 point Likert scale in which a high score indicated greater activity.

*Lockdown compliance*. To measure compliance with lockdown restrictions participants rated “How compliant are you with the current lockdown restrictions?” using a 5 point Likert scale in which 5 indicated greater compliance.

*Change of routine*. Finally, participants also used a 5 point Likert scale to rate to what extent they agreed that: "My daily routine has changed a lot as a result of the Covid-19 lockdown? Here, a high score indicated greater agreement.

#### Data analysis

The analysis strategy replicated that used in Ogden (2020). The main dependant variables, POTJ-day POTJ-week and POTJ-8 month were ordinal scales and therefore non-parametric analyses were conducted. Kruksal-Wallis tests and Mann Whitney U tests were used to establish the effect of age, gender, personal risk, shielding status and cohabitation status on POTJs. In this analysis, as in Ogden (2020) [1], age was classified into three groups; young adults (25 years and under), middle aged adults (26–59 years) and older adults (aged 60 years and over). To assess the relationship between the passage of time and measures of affect (DASS-21 depression, anxiety and stress scores, satisfaction with social interaction), task load (NASA-TXL scores and rating of physical activity), conformity and age, Spearman’s correlations were conducted. Finally, to assess whether these factors were predictive of POTJ’s, separate ordinal logistical regression analyses were conducted for POTJ-day and POTJ-week and POTJ-8 months.

## Results

### The passage of time

**Fig 1:**
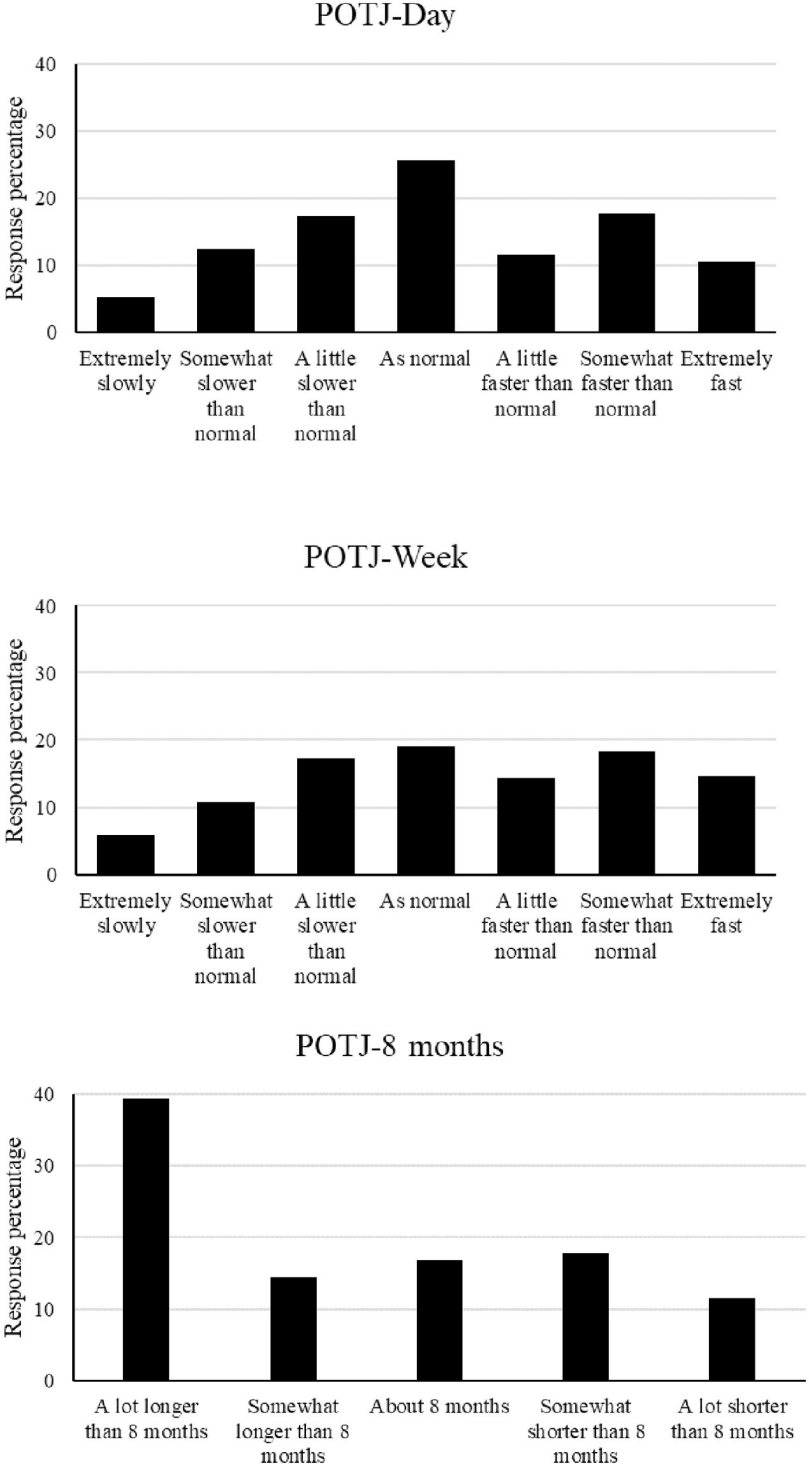
The frequency of responses for each Likert point for the day judgement (upper panel), week judgment (middle panel) and 8-month judgment (lower panel).

Fig 1 shows the distribution of responses for the day (upper), week (middle) and 8 months (lower) passage of time judgments. Examination of Fig 1 suggests that distortion to time was prevalent during the period of study. For POTJ-day, only 26% of participants reported that time was passing at a normal rate, the figure was 19% for POTJ-week. Instead, for POTJ-day 35% of participants reported that time passed more slowly than normal and 39% reported than time passed more quickly than normal. For POTJ-week, 34% of participants reported that time passed more slowly than normal and 47% reported than time passed more quickly than normal. For POTJ-8 months, 17% of participants reported feeling like it was 8 months since the start of the first lockdown, 54% reported that it felt longer than 8 month and 29% reported that it felt shorter than 8 months.

### The effect of demographic factors on POTJ

Table 1 shows mean passage of time judgments expressed as a function of age group, gender, personal risk, occupation and cohabitation status. Table 2 shows analysis of this data using Kruksal-Wallis tests and Mann Whitney U tests. Examination of Table 2 confirms no significant effects of age group, gender, perceived personal risk of covid, cohabitation status and shielding status on any measure of POTJ.

**Table 2:** Outcomes of the analysis of the effect of demographic factors on POTJ’s.

*Variable*	POTJ-Day		POTJ-Week		POTJ- 8 month	
	Statistic	p	Statistic	p	Statistic	p
*Age group*	.11	.95	4.09	.13	1.75	.42
*Gender*	71005.50	.64	71963.50	.84	69915.00	.38
*Perceived risk*	.43	.81	.26	.88	.77	.68
*Cohabitation status*	24695.00	.17	27218.00	.92	25612.00	.35
*Shielding status*	27288.00	.12	22711.50	.06	29334.50	.55

### Factors relating to and predictive of POTJ

Table 3 shows correlation coefficients for the relationships between POTJs and measures of affect, task load, social satisfaction, physical activity, change in daily routine and compliance with restrictions. There were significant positive relationships between satisfaction with social interaction and POTJ-day, week and 8 months. Greater satisfaction with social interaction was therefore associated with a faster POTJ-day and POTJ-week and a shorter perception of 8 months. There was a significant negative relationship between depression and POTJ-day and POTJ-8 months. Greater depression was therefore associated with a slower day and a longer 8 months. There was also a significant negative relationship between stress and POTJ-day and POTJ-8 months with greater stress being associated with a slower day and a longer 8 months. Finally there was also a significant positive relationship between POTJ-8 months and change of life and a significant negative relationship between POTJ-8 months and anxiety. Greater anxiety and greater change in life were associated with a longer perception of the 8 months since the first lockdown began.

**Table 3:** Correlation coefficients between POTJs, age, measures of affect, load, compliance and change to life.

	POTJ- Day	POTJ-Week	POTJ-8 months
*Age*	.01	-.03	.01
*Social satisfaction*	.13**	.14**	.21**
*Change to daily life*	.05	.04	-.16**
*Level of physical Activity*	.07	.05	.08
*Depression*	-.12**	-.07	-.22**
*Anxiety*	-.03	-.04	-.14**
*Stress*	-.08*	-.01	-.21**
*Task-load*	.06	.06	.05
*Compliance*	-.04	-.04	.04

* = *p* < .05,

** = *p* < .001.

Ordinal regression with proportional odds was conducted to establish the effect of demographic factors, measures of affect, task-load and the other measured variables on POTJ-day, POTJ-week and POTJ-8 months separately. Table 4 shows the odds ratios for each variable with 95% confidence intervals.

**Table 4:** Wald, odds ratios and 95% confidence intervals from the ordinal regressions for POTJ-day, POTJ-week and POTJ-8 months.

		*POTJ-day*			*POTJ-week*			*POTJ- 8 month*		
		*Wald*	*Odds Ratio*	*95% CI*	*Wald*	*Odds Ratio*	*95% CI*	*Wald*	*Odds Ratio*	*95% CI*
*Age*		.91	.99	.98–1.01	.83	.99	.98–1.01	.04	1.00	.99–1.02
*Gender*	Female (reference)									
	Male	.004	1.009	.77–1.33	.05	1.03	.78–1.36	.04	1.03	.77–1.37
*Cohabitation status*	Cohabiting (reference)									
	Alone	1.72	1.34	.86–2.09	.004	.99	.64–1.53	1.62	.74	.47–1.18
*Perceived greater risk*	Unsure (reference)									
	No	.02	1.03	.67–1.57	.05	1.05	.69–1.60	.06	1.08	.61–1.89
	Yes	.02	1.04	.61–1.79	.01	1.02	.60–1.76	.37	1.15	.74–1.79
*Self isolating*	No (reference)									
	Yes	5.46*	1.70	1.09–2.66	6.14*	1.76	1.13–2.74	.04	1.05	.66–1.66
*Employment status*	Furloughed (Reference)									
	Employed full time	.34	.83	.43–1.57	.15	.88	.46–1.67	.02	.95	.49–1.88
	Employed part time	.69	.75	.38–1.48	1.16	.69	.35–1.36	2.43	.56	.27–1.16
	Unemployed looking for work	.02	.88	.32–2.66	1.61	.51	.18–1.45	.06	1.15	.38–3.45
	Unemployed not looking for work	.48	.63	.17–2.31	2.92	.32	.09–1.81	3.82	3.75	1.00–14.14
	Retired	1.38	.52	.17–1.55	1.21	.55	.19–1.66	.42	.68	.22–2.16
	Student	1.67	.69	.39–1.22	.55	.81	.46–1.43	.27	1.17	.64–2.15
	Disabled	.71	45	.07–2.89	.95	.40	.06–2.54	1.58	.20	.02–2.45
*Socialisation satisfaction*		7.65**	1.78	1.05–1.32	10.87**	1.21	1.08–1.36	9.00*	1.20	1.07–1.35
*Depression*		4.15*	.96	.93–1.00	4.68*	.96	.93–1.00	5.03*	.96	.92–1.00
*Anxiety*		.03	1.00	.96–1.05	3.74	1.04	1.00–1.05	.57	1.02	.77–1.37
*Stress*		.02	1.00	.96–1.05	.04	1.03	.96–1.07	1.34	.97	.93–1.02
*Task-load*		2.48	1.03	.99–1.07	2.27	1.03	.78–1.36	.17	.99	.96–1.03
*Physical activity*		.30	1.03	.92–1.16	.72	1.05	.94–1.18	.47	1.04	.93–1.17
*Change of routine*		.13	.98	.87–1.10	.32	.97	.86–1.09	13.68**	.80	.71-.90
*Compliance*		2.53	.90	.79–1.03	1.32	.93	.82–1.06	.47	1.05	.92–1.20

* = *p* < .05,

** = *p* < .001.

#### POTJ-day

The model was a statistically significant, χ^2^(21) = 41.10, *p* = .005 fit for the data, with pseudo R squared values of.02 -.05. There were three significant predictors of POTJ-day; satisfaction with social interaction, depression and shielding status. A faster passage of time was associated with greater social satisfaction, lower depression and not shielding.

#### POTJ-week

The model was a statistically significant, χ^2^(21) = 46.78, *p* = .001 fit for the data, with pseudo R squared values of.02 -.06. As for POTJ-day, there were three significant predictors; satisfaction with social interaction, depression and shielding status. A faster passage of time for the week was associated with greater social satisfaction, lower depression and not shielding.

#### POTJ-8 month

The model was a statistically significant, χ^2^(21) = 94.45, *p* < .001 fit for the data, with pseudo R squared values of.03 -.11. There were three significant predictors of POTJ-8 months; satisfaction with social interaction, depression and the extent to which life had changed as a result of lockdown. The 8 months since lockdown feeling short was associated with lower depression, greater social satisfaction, less change of life.

Together these findings highlight the consistent influence of social satisfaction and depression on the experience of time over long and short epochs. Shielding only appears to influence the experience of time over relatively short epochs (day and week) whereas overall change to life affects the experience of time over long epochs (8 months).

## Discussion

This study examined the experience of the passage of time during England’s second national lockdown in response to the covid-19 global pandemic. In particular, the study sought to establish the prevalence of distortions to the passage of time and the factors which predicted peoples experience of time during the second English lockdown.

The results show that distortion to the passage of time was widespread during the second lockdown. Over 70% of people experienced time passing at a speed different to normal during the day. Similarly, over 80% of people experienced time passing at a speed different to normal during the week. The prevalence of distortion to the passage of time during the second English lockdown was similar to that observed during the first lockdown [1]. Greater experience with lockdown does not therefore appear to reduce the experience of distortion to time during the day and the week. Whilst the distortion to the passage of time during the relatively short epochs of the day and the week was reasonably well spread, the experience of time across the 8 months of lockdown was highly skewed towards a slowing. Only 17% of participants reported that the 8 months since lockdown first began felt like 8 months. However, 54% reported that the period felt longer than 8 months. Therefore, despite the fact that the days and the weeks pass more quickly than normal for many, the whole of lockdown seemed longer than normal for the majority. This perhaps indicates that the perception of the passage of time during a long epoch is not simply based on some sort of averaging of recent shorter epochs. One possibility is that responses to POTJ-8 months were based on retrospective memory entries during the 8 month period whereas responses to POTJ-day and POTJ-week were based on recent recollections of temporal experience. Increased memory load is known to lengthen retrospective judgments of duration; greater memory representations are associated with longer perceived durations [32]. The 8 months since the start of the first lockdown have been highly atypical for most people. The continuous movement in and out of different restrictions, coupled with profound changes to the work, home and social lives of many make it likely that there has been a substantive increase in retrospective memory load during this 8 month period. This increased memory load may therefore explain why the majority of people experienced a long 8 months since the first lockdown despite 40% of people experiencing faster than normal weeks and days.

The speed at which time passed was predicted by a number of factors. For POTJ-day and POTJ-week, the relative speed of the passage of time was predicted by satisfaction with social interaction, depression and shielding status. For both judgments, a faster passage of time was associated with greater levels of satisfaction with social interaction, lower levels of depression and not shielding. A slower passage of the day and the week was therefore associated with lower levels of social satisfaction, greater depression and shielding. The relative duration of the POTJ-8 month was predicted by satisfaction with social interaction, depression and the extent to which life had changed as a result of covid-19. A shorter perception of the 8 months since lockdown began was associated with greater levels of satisfaction with social interaction, lower levels of depression and lower levels of change to daily life. A longer perception of the 8 months since lockdown began was therefore associated with lower levels of social interaction, greater depression and greater change to daily life. Age, gender, occupation, personal perceived risk, task-load, stress, anxiety, physical activity, cohabitation status, and compliance with lockdown restrictions were not predictive of the speed at which time passed during the day, week or 8 months.

Comparison of these findings with those reported in Ogden (2020) [1] suggest that despite similarities in the rate of temporal distortion in the first and second UK lockdowns, there were some differences in the factors which influenced the relative speed of time during the two lockdowns. In particular, age, task-load and stress, which were predictors of time experience during the first lockdown did not influence time experience in the second lockdown. The absence of effects of stress and task load perhaps reflect greater adaptation to the lockdown restrictions. At the timing of Ogden’s (2020) [1] survey, the majority of people had very limited experience of working from home and many were additionally trying to balance this with the demands of home schooling. During the second lockdown, schools remained open, significantly reducing the task demands on people working at home with school age children. Similarly, as working remotely has become more normal and many people have reported experiencing it as beneficial and even preferable to working in the office [29]. Interestingly, whilst self-reported task-loads were comparable in the first lockdown (M = 17.43, SD = 3.76) and the current study (M = 18.50, SD = 3.66) there was a reduction in mean stress from the first lockdown [1] in which on 58.80% of people reported normal stress levels and 14.90% reported severe stress levels to the current study in which 83.50% of people reported normal stress levels and 0% reported severe stress. Therefore, these factors may also have contributed to reduced effect of stress and task-load on time experience.

The absence of an effect of age on time experience during the second lockdown suggests that as the pandemic has progressed, time experience became relatively similar across adults of all generations. This contrasts with observations from the first lockdown during which increased age was associated with a slowing of the passage of time. During the first lockdown, it was speculated that time passed more slowly for elderly than younger individuals because they were disproportionately impacted by the virus. This included being at greater risk of mortality from the virus [33] and therefore more likely to avoid face to face social interaction whilst also potentially being less able to utilize technology to maintain social interaction [34]. The absence of an effect of age on time distortion during the second lockdown mirrors the findings of studies conducted prior to lockdown [8]. These have demonstrated that during normal life (i.e. pre lockdown) elderly and younger people experience the passage of time comparably. The absence of an effect of age during the second lockdown perhaps therefore suggests that lockdown is now becoming “normal” for many.

The only consistent predictor of temporal experience during the first and second lockdown was satisfaction with social interaction, with greater satisfaction being associated with a faster passage of time. The enduring influence of social interaction on time experience during both lockdowns likely reflects the consistent effects of both lockdowns on the ability to socialise. Both lockdowns prevented indoor and outdoor mixing of households and therefore severely limited opportunities to engage face-to-face with others. Isolation can increase negative affect and loneliness [28, 35]. Indeed, repeated and extended periods of social isolation are associated with reduced mental wellbeing and increased depression and anxiety [36–38]. The associated emotional effects of social isolation therefore support previous suggestions that negative affect results in a slowing of time during daily activity [8, 39, 40].

As the pandemic has progressed its effect on mental health and wellbeing have become more apparent. Studies examining the effect of lockdown on mental health show significant increases in depression as the pandemic has progressed [23–27]. In the USA, depression rates have increased threefold on pre-pandemic levels [23] and in the UK levels of depression have been found to exceed population norms during lockdown [24]. However, despite these significant changes in mental health and wellbeing, previous studies examining temporal experience during lockdown have failed to observe a relationship between depression and distortion to the passage of time [1–3]. This had been thought to be because other related variables, which were unique to the lockdown restrictions e.g. social satisfaction, were better predictors of time experience than more general measures of affect (e.g. depression) [1]. However, the association between depression and the passage of time in the current study perhaps reflects the increased importance of depression in determining temporal experience because of the growing strain on mental wellbeing as the pandemic has progressed. Indeed, depression levels during the current lockdown (M = 8.57 SD = 5.94) are greater than those reported during the first lockdown (M = 6.40, SD = 5.10). This suggests that the effect of mental health conditions on time experience may take a number of months to become apparent.

Finally, although shielding status was found to be predictive of the passage of the passage of time during the second lockdown, it is important to note that this variable was not assessed in earlier studies. It is therefore impossible to know whether the effect of shielding was present from the start of lockdown or whether it emerged as the lockdown progressed. However, as shielding represents the most extreme form of social and physical distancing, the potential for isolation and the associated effects on mental health are arguably greater and it seems likely that the same affective mechanisms may contribute towards its slowing effect on the passage of time.

### Limitations

This study demonstrates the changing influence of affective, health and task-based factors of time experience throughout lockdown. These variables were selected to enable direct comparison between Ogden’s (2020) [1] UK study conducted during the early part of the UK’s first lockdown. However, as was the case in Ogden (2020) [1], these variables only account for a small proportion of the variance in the speed at which time was felt to pass. This suggests that other factors are also determining temporal experience. Future research should therefore seek to establish the influence of a wider set of variables on temporal experience.

A further limitation is that the current study introduced two new variables which were not studied during the first lockdown; compliance with restrictions and shielding status. It is not therefore possible to compare the influence of these variables across the two lockdowns. Furthermore, although the current study found no association between compliance with covid restrictions and the subjective speed of time during lockdown, it is possible that there were biases in the reporting of compliance. The self-reported levels of compliance were high, with 41% of people reporting being extremely compliant and 38% reporting being somewhat compliant. However, this may reflect social pressure to be seen to comply rather than actual levels of compliance. It is therefore possible that a more reliable measure of compliance may produce different findings.

## Conclusion

The results of this study suggest that distortions to the passage of time were endemic during England’s second national lockdown. Despite significant experience with social and physical distancing, lockdown restrictions were still distorting the passage of time for the majority of people. This suggests that fundamental changes to the structure of daily life can distort the passage of time in the short and long term. During the second lockdown, the passage of time was affected by satisfaction with social interaction, depression, shielding status and the extent to which life had changed as a result of lockdown. Collectively, these findings confirm previous suggestions that negative affect is associated with a slowing of the passage of time during lockdown [1–3] and during normal life [8, 38, 39]. However, the changes in predictive factors between the first and second lockdowns demonstrate the importance of examining the long-term effects of the pandemic on perception and cognition.

## Supporting information

S1 Data (SAV)
